# Supplementary material for: Structural Rationalization of IPMK Inhibitor Potency
Source: J Med Chem. 2025 Nov 14;68(22):24316–25. doi: 10.1021/acs.jmedchem.5c02314 (PMC12670395; doi:10.1021/acs.jmedchem.5c02314)
Supplement: Supplementary file 1 [file jm5c02314_si_001.pdf]

Supporting Information for Publication

Structural rationalization of IPMK inhibitor potency.

Huanchen Wang[1]*, Stephen B. Shears[1] and Raymond D. Blind[2,3]*

[1]Molecular and Cellular Biology Laboratory, National Institute of Environmental Health Sciences, NIH, Research Triangle Park, NC 27709, USA.
[2]Department of Medicine, Division of Diabetes, Endocrinology & Metabolism, Vanderbilt University Medical Center, Nashville, TN 37232, USA.
[3]Departments of Biochemistry & Pharmacology, Vanderbilt University School of Medicine, Nashville, TN 37232, USA.

*Correspondence:
huanchen.wang@nih.gov
ray.blind@vanderbilt.edu

Table of Contents for Supporting Information:

Supporting Table S1, Crystallography collection, refinement statistics …………………….. Page S3-S4
Supporting Table S2, Ordered waters, residue contacts ..……………………………………. Page S5
Supporting Figure S1, Superposed Compound 1 site for IP3K-A and IP6K1 ………………. Page S5
Supporting Table S3, Molecular Formula SMILES strings, IC_{50} data ………………………. Page S6

Supplementary Table S1, Data collection and refinement statistics

PDB Accession Codes	**8V6W**	**8V6X**	**8V6Y**	**8V6Z**	**8V70**	**8V71**	**8V72**
Compound	1	2	3	4	6	7	8
Space group	$P4_22_12$	$P4_22_12$	$P4_22_12$	$P4_22_12$	$P4_22_12$	$P4_22_12$	$P4_22_12$
Cell parameters a, c (Å)	77.7, 84.8	78.2, 84.3	78.4 85.2	77.9, 85.6	78.1, 84.8	78.3, 85.1	77.9, 85.0
Resolution (Å)*	1.95 (1.98)	1.75 (1.78)	1.7 (1.73)	1.85 (1.88)	1.85 (1.88)	1.70 (1.73)	2.0 (2.03)
Rmeas*	0.108 (0.816)	0.069 (0.693)	0.042 (0.793)	0.119 (0.819)	0.092 (0.813)	0.056 (0.783)	0.127 (0.713)
I/σ I*	19.3 (2.4)	42.5 (5.0)	65.2 (4.0)	20.4 (2.2)	20.9 (2.4)	52.0 (4.8)	20.8 (3.6)
Completeness (%)*	99.7 (100.0)	99.7 (99.9)	99.3 (99.9)	99.1 (98.6)	99.7 (100.0)	99.5 (100.0)	99.9 (100.0)
Redundancy*	10.1 (10.7)	15.2 (14.8)	14.2 (14.3)	11.4 (8.6)	7.8 (7.2)	16.4 (16.5)	12.5 (11.1)
Refinement							
No. reflections*	19354 (1802)	26756 (2566)	29501 (2813)	22642 (2061)	22620 (2002)	29485 (2863)	18216 (1751)
R_{work}*	0.162 (0.193)	0.189 (0.233)	0.144 (0.190)	0.173 (0.231)	0.190 (0.248)	0.183 (0.226)	0.172 (0.208)
R_{free}*	0.228 (0.271)	0.242 (0.275)	0.191 (0.271)	0.229 (0.314)	0.239 (0.269)	0.230 (0.330)	0.243 (0.309)
No. atoms							
Protein	1941	1963	1992	1969	1958	1983	1968
ligands	22	23	20	22	25	24	30
solvent	110	179	182	157	130	163	134
R.M.S. deviations							
Bond length (Å)	0.007	0.007	0.009	0.006	0.006	0.007	0.006
Bond angle (°)	1.24	1.14	1.39	1.01	1.11	1.14	1.06
B-factors (Å^2)							
Protein	33.5	38.2	25.9	26.1	30.7	23.2	33.1
ligand	26.1	27.9	17.7	20.4	29.2	14.6	31.7
solvent	41.9	53.9	38.4	36.8	48.2	38.1	45.4
Ramachandran							
favored (%)	99.2	97.4	99.1	97.4	96.5	99.1	98.7
allowed (%)	0.4	2.6	0.9	2.6	2.6	0.9	1.3
outliers (%)	0.4	0.0	0.0	0.0	0.9	0.0	0.0
Rotamer outliers (%)	1.4	0.0	0.9	0.0	0.9	0.9	0.0
Clash score	3.1	4.3	3.5	1.8	2.8	2.8	2.5

*The numbers in parentheses are given for the highest-resolution shells, rotamer outliers and clash scores were calculated using Phenix, authors will release the atomic coordinates upon article publication

Supplementary Table S1, continued Data collection and refinement statistics

PDB Accession Codes	**8V73**	**8V74**	**8V75**	**8V77**	**8V78**	**8V79**	**8V76**
Compound	9	10	11	12	13	14	15
Space group	$P4_22_12$	$P4_22_12$	$P4_22_12$	$P4_22_12$	$P4_22_12$	$P4_22_12$	$P4_22_12$
Cell parameters a, c (Å)	77.8, 84.9	77.8, 85.5	77.8, 85.3	77.8, 85.3	78.2, 85.3	77.8, 85.0	78.5, 85.5
Resolution (Å)*	1.9 (1.93)	1.85 (1.88)	1.85 (1.88)	1.95 (1.98)	1.95 (1.98)	1.95 (1.98)	1.95 (1.98)
Rmeas*	0.113 (0.958)	0.110 (0.912)	0.085 (0.851)	0.110 (0.864)	0.111 (0.661)	0.094 (0.816)	0.085 (0.969)
I/σ I*	22.8 (2.5)	29.9 (3.3)	36.9 (3.6)	25.9 (3.0)	23.7 (3.4)	25.9 (2.2)	33.8 (3.1)
Completeness (%)*	99.7 (100.0)	99.9 (99.6)	99.8 (99.9)	100.0 (100.0)	99.8 (99.9)	97.3 (90.9)	98.8 (98.0)
Redundancy*	14.2 (11.4)	18.6 (16.3)	17.8 (15.5)	14.2 (12.7)	12.7 (10.4)	14.8 (8.8)	17.6 (14.3)
Refinement							
No. reflections*	20844 (1851)	22992 (2215)	22933 (2213)	19555 (1821)	19526 (1893)	18676 (1435)	19620 (1885)
R_{work}*	0.160 (0.213)	0.158 (0.202)	0.159 (0.231)	0.161 (0.200)	0.171 (0.208)	0.175 (0.221)	0.161 (0.263)
R_{free}*	0.229 (0.258)	0.217 (0.266)	0.221 (0.299)	0.217 (0.303)	0.221 (0.243)	0.241 (0.291)	0.217 (0.337)
No. atoms							
Protein	1953	1957	1948	1965	1965	1947	1965
ligands	27	26	31	27	29	30	27
solvent	146	171	173	132	138	128	132
R.M.S. deviations							
Bond length (Å)	0.007	0.006	0.007	0.006	0.006	0.007	0.006
Bond angle (°)	1.26	1.09	1.09	1.13	1.09	1.18	1.13
B-factors ($Å^2$)							
Protein	28.8	26.4	26.8	28.4	28.8	34.4	28.4
ligand	26.5	29.9	21.9	27.2	27.1	26.7	27.2
solvent	35.0	35.4	38.6	40.7	42.8	44.5	40.7
Ramachandran							
favored (%)	98.7	97.9	99.1	98.3	99.1	97.9	98.3
allowed (%)	0.9	2.1	0.9	1.7	0.9	2.1	1.7
outliers (%)	0.4	0.0	0.0	0.0	0.0	0.0	0.0
Rotamer outliers (%)	0.9	0.5	0.0	0.0	0.0	0.0	0.0
Clash score	3.9	2.1	2.6	2.6	2.8	2.8	2.6

*The numbers in parentheses are given for the highest-resolution shells, rotamer outliers and clash scores were calculated using Phenix, authors will release the atomic coordinates upon article publication

Supplementary Table S2, Ordered Water and Residue Contacts for each structure

Inhibitor assigned number	PDB Codes	Number of hinge region polar contacts (<3.5Å)	Moiety A Water1	Moiety A Water2	Moiety B Water3	Moiety B additional VDW contact residues	Moiety B Polar contact residues	IC_{50} (nM)
1	8V6W	3	-	-	-			12.8
2	8V6X	2	+	-	-			8.1
3	8V6Y	3	+	-	-			29
4	8V6Z	4	+	+	-			1.9
6	8V70	4	+	+	-			3.8
7	8V71	4	+	+	-	R182		2.5
8	8V72	4	+	+	-			3.3
9	8V73	4	+	+	-	R182		11
10	8V74	4	+	+	-			3.5
11	8V75	4	+	+	+	G180, M181, R182	R182	1.0
12	8V77	4	+	+	-	D144		3.4
13	8V78	4	+	+	-	G180, M181	G180, M181	9.5
14	8V79	4	+	+	-	D144, L179		2.8
15	8V76	4	+	+	+	D144, G180, M181		1.13

Supplementary Figure S1, Superposed Compound 1 binding site onto IP3K-A and IP6K1

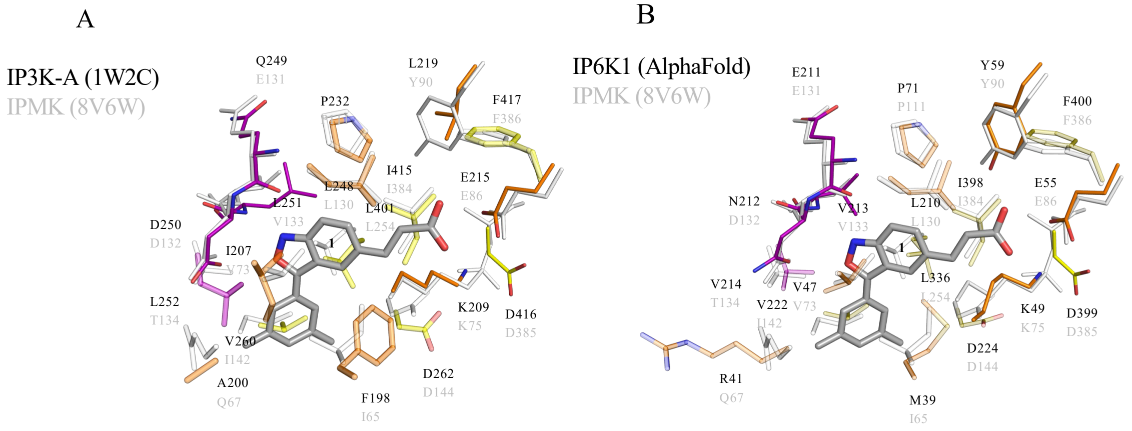

Supplementary Figure S1. *The amino acids surrounding compound 1 in the IPMK ATP-binding active site are largely conserved with IP3K-A and IP6K1.* Model of Compound 1 (dark gray sticks) from the crystal structure reported here (8V6W) docked into **A.** PDB: 1W2C (IP3K-A and **B**. an Alpha-fold predicted model of IP6K1, superimposed with the IPMK (8V6W) complex structure (light gray sticks). *These data suggest the compound 1 binding site is largely conserved between these enzymes, with some noted differences.*

Supplementary Table S3

Compound_ID	UNC_id	SMILES	IPMK IC50 (nM)	IP6K1 IC50 (nM)	IP3K-A IC50 (nm)
1	UNC7437	CC1=CC(C)=CC(C2=C3C=C(/C=C/C(O)=O)C=CC3=NO2)=C1	12.8 ±2nM	146 ±23nM	8000 ±1500nM
2	UNC7789	CC1=CC(C)=CC(C2=C3C=C([C@@H]4[C@@H](C(O)=O)C4)C=CC3=NO2)=C1	8.1 ±2	45 ±7	n/d
3	UNC7438	OC(C1=CC2=C(C3=CC(C)=CC(C)=C3)ON=C2C=C1)=O	29 ±5	84 ±24	n/d
4	UNC7854	CC1=CC(C)=CC(C2=C3C=C(C4=NN=NN4)C=CC3=NO2)=C1	1.9 ±0.2	24 ±6	n/d
6	UNC7782	C1(C2=NN=NN2)=CC3=C(C4=CC=CC=C4)ON=C3C=C1	3.8 ±0.4	38 ±8	n/d
7	UNC7855	FC(C(C=C1)=CC=C1C2=C3C=C(C4=NN=NN4)C=CC3=NO2)(F)F	2.5 ±0.3	23 ±4	n/d
8	UNC7891	FC(OC(C=C1)=CC=C1C2=C3C=C(C4=NN=NN4)C=CC3=NO2)(F)F	3.3 ±0.4	59 ±14	n/d
9	UNC7890	C1(C2=NN=NN2)=CC3=C(C4=CC=C(OC5=CC=CC=C5)C=C4)ON=C3C=C1	11 ±2	1300 ±330	n/d
10	UNC8787	O=C(C1CC1)NC(C=C2)=CC=C2C3=C4C=C(C5=NN=NN5)C=CC4=NO3	3.5 ±0.4	36 ±6	n/d
11	UNC9021	O=C(C1CC1)NC2=CC=CC(C3=C4C=C(C5=NN=NN5)C=CC4=NO3)=C2	1.0 ±0.2	51.8 ±5	n/d
12	UNC9120	C1(C2=NN=NN2)=CC3=C(C4=CC(OC5CCNCC5)=CC=C4)ON=C3C=C1	3.4 ±0.5	30 ±6	n/d
13	UNC9107	ClC(C=C(Cl)C=N1)=C1OC2=CC=CC(C3=C4C=C(C5=NN=NN5)C=CC4=NO3)=C2	9.5 ±2	19.0 ±4	n/d
14	UNC9750	C1(C2=NN=NN2)=CC3=C(C4=CC(C5CCN(C6CCC6)CC5)=CC=C4)ON=C3C=C1	2.8 ±0.6	155 ±23	n/d
15	UNC9022	O=C(C1CCCC1)NC2=CC(C3=C(C=C(C4=NN=NN4)C=C5)C5=NO3)=CC=C2	1.13 ±0.2	40.1 ±4	n/d
